# Hyperthermia Induces Apoptosis of 786-O Cells through Suppressing Ku80 Expression

**DOI:** 10.1371/journal.pone.0122977

**Published:** 2015-04-22

**Authors:** Defeng Qi, Yuan Hu, Jinhui Li, Tao Peng, Jialin Su, Yun He, Weidong Ji

**Affiliations:** 1 Department of Urology, Minimally Invasive Surgery Center, The First Affiliated Hospital of Guangzhou Medical University, Guangdong Provincial Key Laboratory of Urology, Guangzhou, China; 2 School of public health, Sun Yat-sen University, Guangzhou, China; 3 The center for translational medicine, The first affiliated hospital, Sun Yat-Sen University, Guangzhou, China; St. Georges University of London, UNITED KINGDOM

## Abstract

Hyperthermia as an anticancer method has been paid increasing attention in recent years. Several studies have shown that hyperthermia can kill tumor cells by inducing apoptosis. However, the underlying molecular mechanisms of hyperthermia-induced apoptosis are largely unknown. To investigate the effects and molecular mechanism of hyperthermia on the apoptosis in renal carcinoma 786-O cells, we firstly examined apoptosis and Ku expression in 786-O cell line treated with heat exposure (42°C for 0-4 h). The results showed that hyperthermia induced apoptosis of 786-O cells, and suppressed significantly Ku80 expression, but not Ku70 expression. Next, we knock-down Ku80 in 786-O cells, generating stable cell line 786-O-shKu80, and detected apoptosis, cell survival and cell cycle distribution. Our data showed higher apoptotic rate and lower surviving fraction in the stable cell line 786-O-shKu80 compared with those in control cells, exposed to the same heat stress (42°C for 0-4 h). Moreover, the results also showed suppression of Ku80 led to G2/M phase arrest in the stable cell line 786-O-shKu80 following heat treatment. Together, these findings indicate that Ku80 may play an important role in hyperthermia-induced apoptosis and heat-sensitivity of renal carcinoma cells through influencing the cell cycle distribution.

## Introduction

Renal cell carcinoma (RCC) is the most common form of kidney cancer and represents more than 90% of the solid malignant masses observed in the kidney [1]. To date, RCC treatment is still a very challenging task due to no effective strategy in treating the late stages of this disease, so it is usually followed by a poor prognosis. Therefore, it is essential to develop more effective therapeutic strategies for RCC.

Hyperthermia is a therapeutic procedure that increases the temperature in body tissues in order to change the function of the cellular structures. In the past two decades, there has been a great interest in application of hyperthermia in conjunction with irradiation or/and chemotherapy in cancer treatment. The promising results from recent clinical trials indicate the effectiveness of hyperthermia treatment as an adjunct to radiotherapy or chemotherapy in treating numerous cancers, including superficial cutaneous tumors, recurrent breast cancer, recurrent malignant melanoma, head and neck squamous cell carcinoma, lymph node metastases, glioblastoma, cervical carcinoma and RCC [2–4]. Hyperthermia has profound effects on tumor cells, involving in the induction of intrinsic programmed cell death pathways such as apoptosis, cell death by mitotic catastrophe and inhibition of cell proliferation by stimulating replicative senescence [5–7]. If the therapeutic temperature ranges from 40 to 45°C, hyperthermia kills tumor cells mainly by inducing apoptosis, whereas hyperthermia with higher temperature results in necrosis [8, 9]. Although heat-induced apoptosis has been recognized, the underlying molecular mechanisms of hyperthermia-induced apoptosis still are largely unknown.

DNA double-strand breaks (DSBs) caused by endogenous (byproducts of cellular metabolism and replication associated errors) and exogenous (ionizing radiation and chemotherapeutic drugs) agents, are the most lethal damage among the different kind of DNA damages as unrepaired DSBs can result in genomic instability, cell death and tumorigenesis [10]. It can be repaired via two major pathways: homologous recombination (HR) and nonhomologous end joining (NHEJ). The NHEJ pathway is regarded as the major pathway for the repair of radiation induced DSBs in mammalian cells [11–15]. One of the main participants in this pathway is the DNA-dependent protein kinase (DNA-PK) that consists of a large catalytic subunit, DNA-PKcs, and a heterodimeric protein named Ku, which is a highly stable protein complex consisting of a 70 kDa and a 86 kDa polypeptide, better known as Ku70 and Ku80 [16–20]. NHEJ is initiated by the DNA repair protein Ku, which recognizes DSBs and recruits additional pathway components to process and repair the damage. Therefore, Ku plays a crucial role for DSBs repair.

The combined effect of heat and radiation is referred to as heat or thermal radiosensitization, which is believed to be caused by an inhibition of repair of radiation-induced DSBs by hyperthermia [21, 22]. A similar effect is observed with many chemotherapeutic drugs [23]. Moreover, it has been reported that defect in or absence of Ku70, Ku80, or DNA-PKcs subunit results in deficiencies in DNA-DSB repair, leading to hypersensitivity to ionizing radiation and anticancer drugs [24–29]. Iharaet al. also showed that hyperthermia suppressed the expression of Ku70 and Ku80 in hybrid cells containing a fragment of human chromosome 8 in scid mouse cells [30]. Therefore, Ku expression is associated with heat, radio- and chemosensitization. However, to our knowledge, there are little studies that investigate whether hyperthermia can affect the expression of Ku in RCC.

According to the research conclusions above, this study was designed to detect the relationship between hyperthermia and Ku expression in renal carcinoma 786-O cells, and investigate the possible underlying molecular mechanisms of hyperthermia-induced apoptosis.

## Materials and Methods

### Cell culture

Human renal carcinoma cell line (786-O) was obtained from Keygen company (Nanjing, China) and cultured in RPMI 1640 medium (Gibco, USA) supplemented with 10% fetal bovine serum (Gibco, USA). 293FT cells (Keygen, Nanjing, China) were grown in Dulbecco's modified Eagle's Medium (DMEM) medium (Gibco, USA) supplemented with 10% fetal bovine serum. All cell cultures were maintained at 37°C in a humidified 5% CO_2_ incubator.

### Heat treatment

The cells were seeded in 25-cm^2^ tissue culture flasks for 24 h to allow exponential growth. Then, the flasks were sealed with parafilm and immersed in a temperature-controlled waterbath at 37°C or 42°C in different time groups (0, 0.5, 1, 2, 4h). After the heat treatment, the flasks were removed from the waterbath and processed immediately.

### Construction of recombinant retroviral vectors and establishment of stable human renal carcinoma 786-O cell lines

Three pairs of effective RNA interference (RNAi) sequences (Table 1) to target Ku80 gene were designed and synthesized. Then each sequence was annealed to form double-stranded DNA and connected to pSUPERretro-puro vector digested by Bgl Ⅱ and Hind Ⅲ. The recombinant vectors were designated as pSUPERretro-puro-shKu80 and identified by sequencing. At the same time, we also constructed the control vector named pSUPERretro-puro-scramble. After the Ku80 shRNA target sequence with the strongest silencing effect was screened, both of the vectors were co-transfected in 293FT cells. Generated virus particles subsequently infected 786-O cells, the positive clones were obtained following puromycin selection. The stable cell lines achieved were correspondingly designated as 786-O-shKu80 and 786-O-scramble.

**Table 1:** RNAi sequences used in this study.

sequences Name	RNAi sequence(5′———3′)	
**shKu80- 1**	F: GATCCCCGGTGGCCATAGTTCGATATGC TTCAAGAGAGCATATCGAACTATGGCCACC TTTTT	R: AGCTTAAAAAGGTGGCCATAGTTCGATATGC TCTCTTGAAGCATATCGAACTATGGCCACCGGG
**shKu80-2**	F: GATCCCCGGCACAGTTGAATGCTGTTGA TTCAAGAGATCAACAGCATTCAACTGTGCC TTTTT	R: AGCTTAAAAAGGCACAGTTGAATGCTGTTGA TCTCTTGAATCAACAGCATTCAACTGTGCCGGG
**shKu80-3**	F: GATCCCCGCTTTGAGGAAGCGAGTAACC TTCAAGAGAGGTTACTCGCTTCCTCAAAGC TTTTT	R: AGCTTAAAAAGCTTTGAGGAAGCGAGTAACC TCTCTTGAAGGTTACTCGCTTCCTCAAAGCGGG
**scramble**	F: GATCCCCGCCAGCTTAGCACTGACTC TTCAAGAGAGAGTCAGTGCTAAGCTGGC TTTTT	R: AGCTTAAAAAGCCAGCTTAGCACTGACTC TCTCTTGAAGAGTCAGTGCTAAGCTGGCGGG

### Western blot analysis

Cultured cells were collected and washed twice with 1ml of PBS. After cracked with total protein lysis buffer, the cells were cooled on ice for 10 min and collected in a centrifuge tube. 50μl of protein sample were mixed with 50μl of SDS loading buffer and incubated for 5 min at 100°C. Then the samples were run on a SDS-PAGE gel, after electrophoresis, the proteins were transferred onto PVDF membrane and detected by immunolabelling with primary and secondary antibodies. In this experiment, we made the β-actin as the internal reference.

### Real-time quantitative PCR

Total RNA was extracted using the Trizol reagent according to the instructions. The RNA purity and concentration were determined by the UV spectrophotometer. CDNA was reversibly transcribed from the extracted total RNA using an MMLV reagent kit (TaKaRa, Japan). The Ku80 and β-actin primer sequences were designed as Table 2. PCR was then carried out as follows: denaturing at 95°C for 20s, 40 cycles of 10s at 95°C, 20s at 58°C and 30s at 72°C.

**Table 2:** Primers used in this study.

Primer Name	Primer sequence(5′———3′)	
**RT-Q-PCR primers**		
**Ku80**	F: CCACCGAGGCACAGTTGAAT	R: TCTGTGCAGCAGACACTGAA
**β-actin**	F: TGGCACCCAGCACAATGAA	R: CTAAGTCATAGTCCGCCTAGAAGCA

### Apoptosis determination by flow cytometry

The stable 786-O cell lines were harvested by centrifugation for 3 min at 1000rpm and were resuspended in binding buffer. Aliquots containing 1 × 10^5^ cells in 190μl of buffer were stained with 10μl of PI solution and with 5μl of Annexin V-FITC (eBioscience, USA) for 10 min at room temperature. Then Flow cytometric analysis was performed using a flow cytometer (BD, USA) to detect the cell apoptosis.

### Colony formation assay

Cell survival was measured using a standard colony forming assay. After heat treatment, cells were seeded onto six-well plates at 500 cells per well. One week later, colonies were fixed with 100% methanol for 15 min and stained with 0.1% crystal violet for 20 min. Microscopic colonies composed of more than approximately 50 cells were counted as having grown from surviving cells.

### Cell cycle analysis

Cells were collected by trypsin method, washed with PBS, fixed overnight at 4°C in 70% ethanol. They were then washed in cold PBS and resuspended in 50μg/mL propidium iodide and RNase A (50μg/mL). The cell suspension was incubated in a 37°C water bath for 1 h and cell cycle distribution was determined by flow cytometry.

### Statistical analysis

All of the experiments were replicated three times. The results are presented as the mean ± standard deviation (SD). Statistical analysis was performed by one-way analysis of variance (ANOVA) followed by LSD multiple comparison using software SPSS 13.0. A value of P<0.05 was considered to be statistically significant and indicated with * in the figures and tables.

## Results

### Ku80 expression in stable cell lines

**Fig 1:**
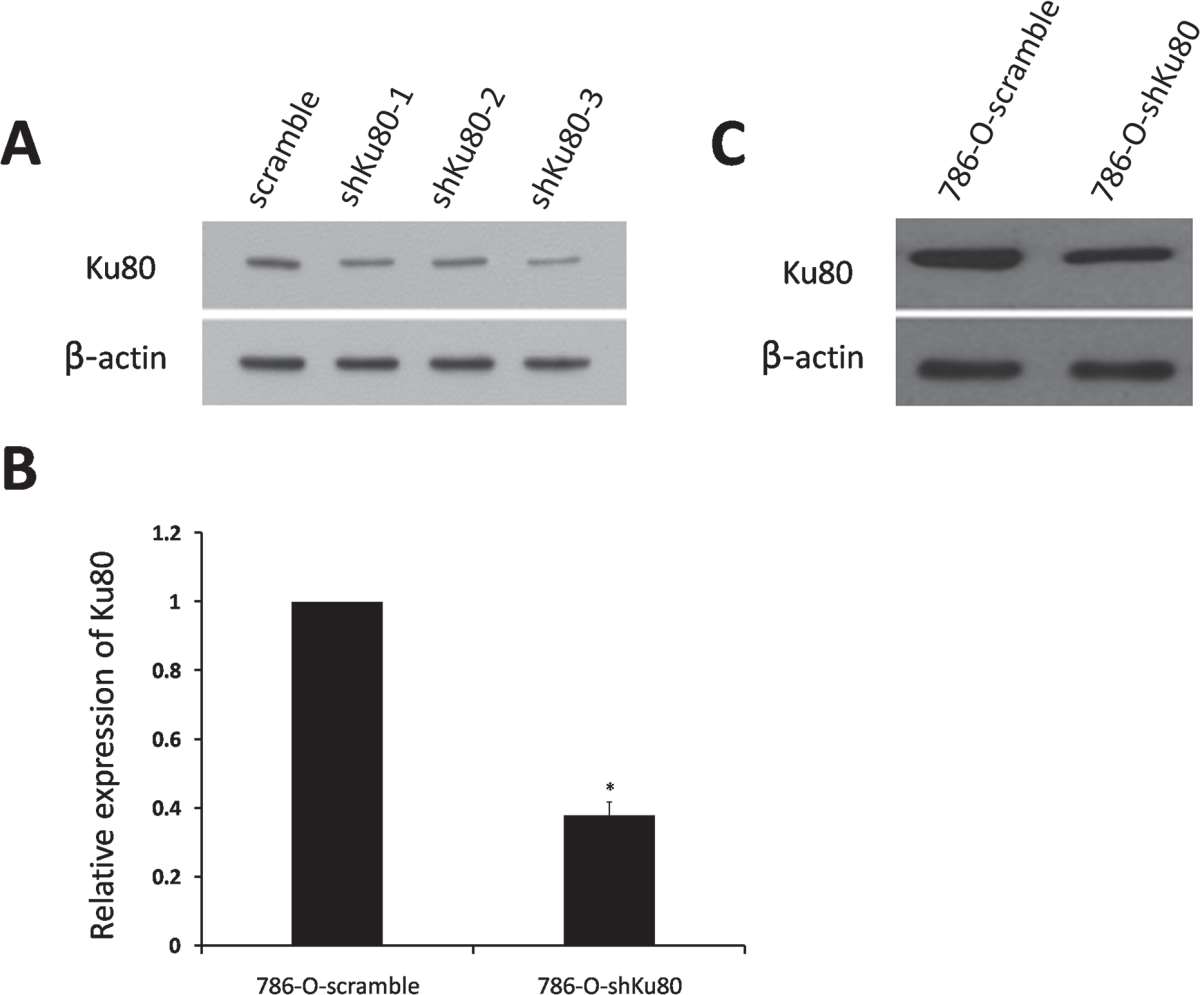
Ku80 expression was measured in the stable 786-O cell lines. **A:** Effect of various shRNAs on Ku80 expression was analyzed by Western blot in 786-O cells. **B:** Ku80 mRNA expression was detected by RT-PCR in the stable 786-O cell lines. **C:** Ku80 protein expression was detected by Western blot in the stable 786-O cell lines. *P<0.05 is considered significant.

In order to construct the pSUPERretro-puro-shKu80 vector, three pairs of effective RNA interference sequences (shKu80-1, shKu80-2 and shKu80-3) to target Ku80 gene were designed and synthesized. To determine the effect of various shRNAs on Ku80 protein expression, we measured the amount of Ku80 by Western blot analysis. Of the three Ku80 shRNAs, shKu80-3 was the most effective in suppressing the expression of Ku80 (Fig 1A). Therefore, the recombinant plasmid vector containing shKu80-3 was screened out for establishing stably knock-down 786-O cell line.

Ku80 expression in stable cell lines was determined by RT-PCR and Western blot analysis. Compared with stable 786-O control cells, the expression of Ku80 mRNA was reduced by 62% in 786-O-shKu80 cells (Fig 1B), which was consistent with the results of Western blot analysis (Fig 1C). The results indicate the stable 786-O cell line with low-expression of Ku80 was successfully generated by retrovirus-mediated method.

### Effect of hyperthermia on apoptosis in 786-O cells

**Fig 2:**
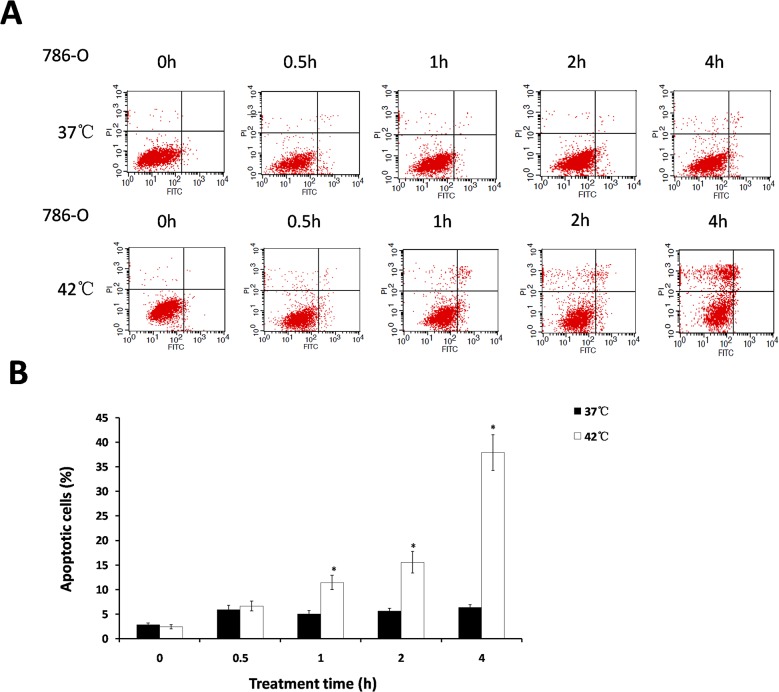
Hyperthermia induces apoptosis in 786-O cells. Cells were exposed to 37°C and 42°C for the indicated amount of time. Apoptotic cells were measured by flow cytometry immediately after heat treatment. **A:** Images showing flow cytometric analysis of apoptosis. **B:** The histogram shows the result from A (%). *P<0.05 compared to control. Results are representative of three independent experiments.

To examine whether hyperthermia induces apoptosis in 786-O cells, we exposed the 786-O cells to 37°C (control) and 42°C (hyperthermia) in different time groups (0, 0.5, 1, 2, 4h) respectively, and performed flow cytometry to determine the level of spontaneous apoptosis. The date showed that no significant change of apoptosis was observed after heating for various times at 37°C. In contrast, the percentages of apoptotic cells significantly increased after treatment for 1h at 42°C, and when cells were exposed to hyperthermia for 1h, 2h and 4h, it was increased by 6.4%, 9.9% and 31.6% compared to control, respectively (Fig 2). These results show that hyperthermia can induce apoptosis in 786-O cells.

### Effect of hyperthermia on Ku expression in 786-O cells

**Fig 3:**
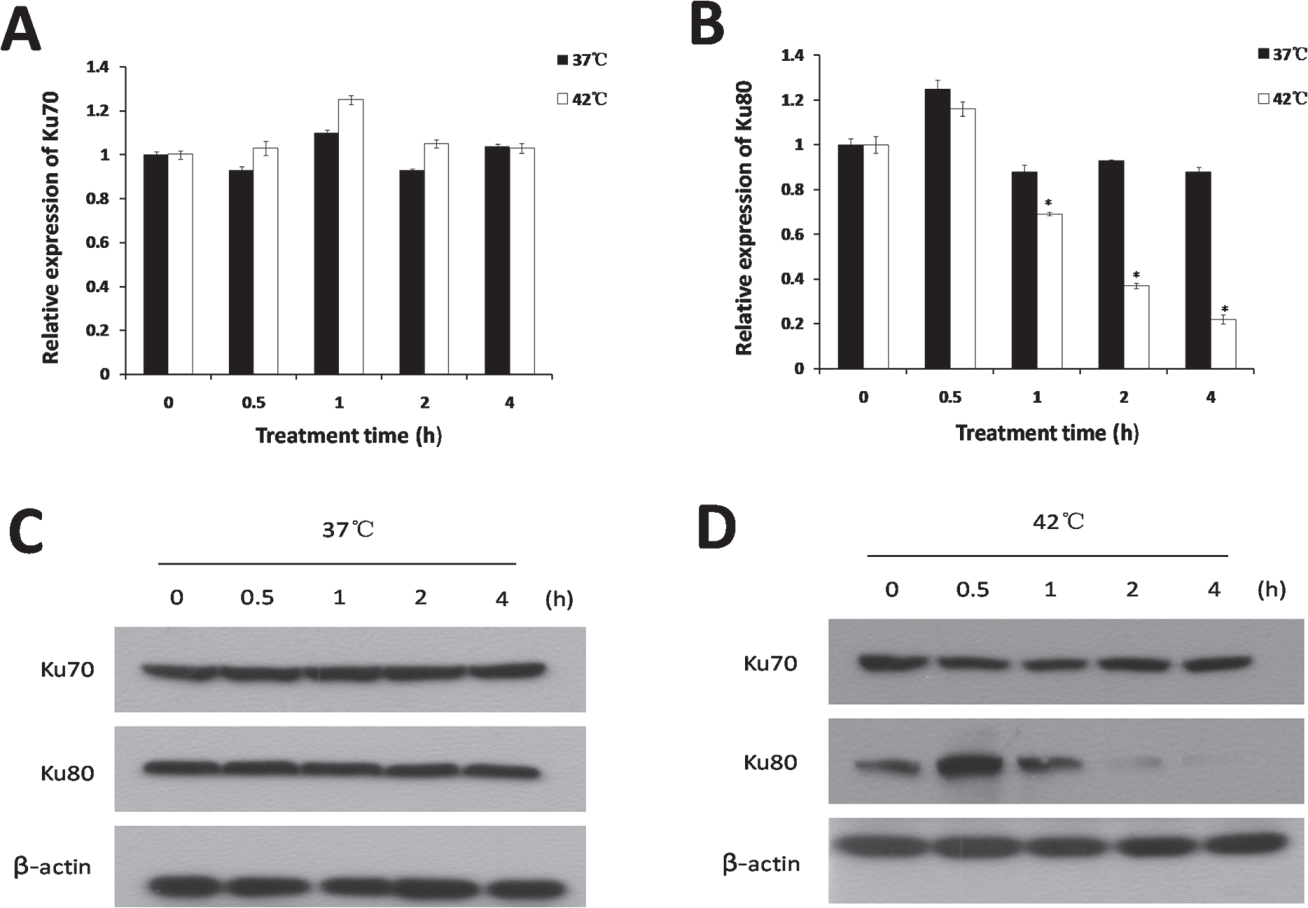
Ku expression was detected in 786-O cells exposed to 37°C or 42°C for the indicated amount of time. **A** and **B:** Ku70 and Ku80 mRNA expression was analysed by RT-PCR. **C** and **D:** Ku70 and Ku80 protein expression was detected by Western blot. *P<0.05 compared to control.

To investigate whether hyperthermia can affect the expression of Ku70 and Ku80, the 786-O cells were also exposed to 37°C and 42°C for five time points respectively, then the expression of Ku70 and Ku80 was detected by RT-PCR and Western blot analysis. The result of RT-PCR (Fig 3A and 3B) indicated that there was no significant difference in the amount of Ku70 mRNA both in the 37°C-treated control cells and 42°C-treated cells during the heating process. In addition, no effect on Ku80 mRNA expression was observed in 786-O cells exposed to 37°C for five time points. However, down regulation of Ku80 mRNA expression was found after hyperthermia (42°C) for 1h, and the reduction of expression was the highest after hyperthermia for 4h. Compared to control, the expression of Ku80 mRNA was decreased by 19%, 56% and 66% after hyperthermia for 1h, 2h and 4h, respectively, suggesting Ku80 expression was suppressed by hyperthermia. On the other hand, we also achieved accordant result with RT-PCR using Western blot analysis (Fig 3C and 3D). According to these findings, we conclude that hyperthermia can inhibit the expression of Ku80.

### Ku80 expression correlates with hyperthermia-induced apoptosis

**Fig 4:**
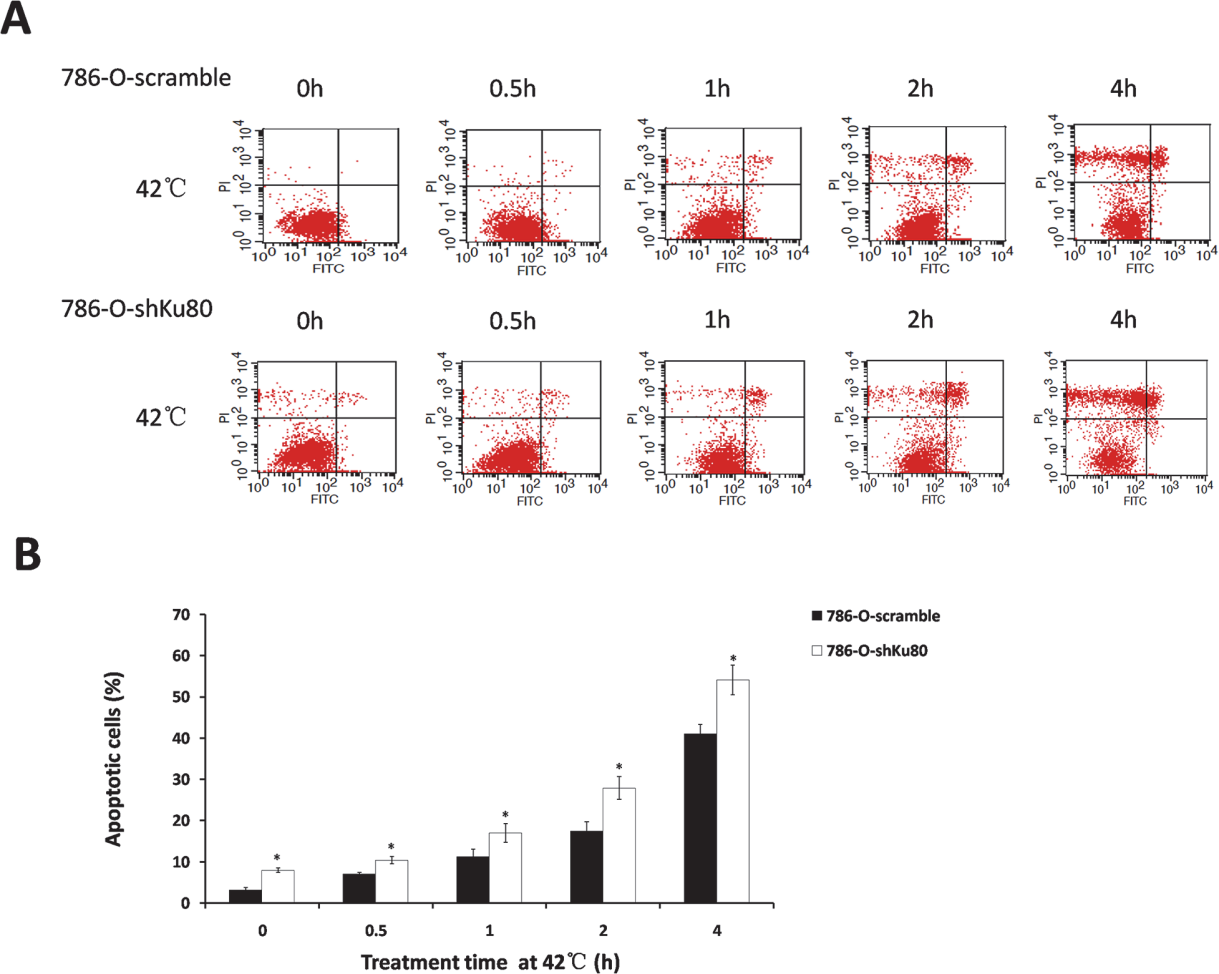
Response of 786-O-shKu80 and 786-O-scramble cells to hyperthermia. Cells were exposed to 42°C for the indicated amount of time. Apoptotic cells were measured by flow cytometry immediately after hyperthermia. **A:** Images showing flow cytometric analysis of apoptosis. **B:** The histogram shows the result from A (%). *P<0.05 compared to control. Each date point is the mean of three independent experiments.

Hyperthermia induced apoptosis with reduction of Ku80 expression in 786-O cells, which made us wonder if the down-regulation of Ku80 expression could trigger apoptosis directly. To test this, 786-O-shKu80 cells with low-expression of Ku80 and 786-O-scramble cells as a control were generated by retrovirus-mediated method and subjected to 42°C for various times. After heat treatment, the cells were harvested and the level of spontaneous apoptosis was detected by flow cytometry. As shown in Fig 4, the apoptotic rates of 786-O-shKu80 cells were significantly higher than 786-O-scramble cells at each time point, and at 1h, 2h and 4h, the apoptotic rate of 786-O-shKu80 cells was increased by 5.7%, 10.4% and 13.1%, respectively. Thus, we can easily attain an important apocalypse that hyperthermia-induced apoptosis can be mediated by Ku80 expression and reduction of Ku80 expression induces apoptosis directly under the condition of hyperthermia in 786-O cells. Furthermore, as the effect of hyperthermia on apoptosis can be enhanced by inhibition of Ku80, down-regulation of Ku80 expression combined with hyperthermia will be a potential strategy for RCC therapy.

### Effect of Ku80 expression on heat-sensitivity

**Fig 5:**
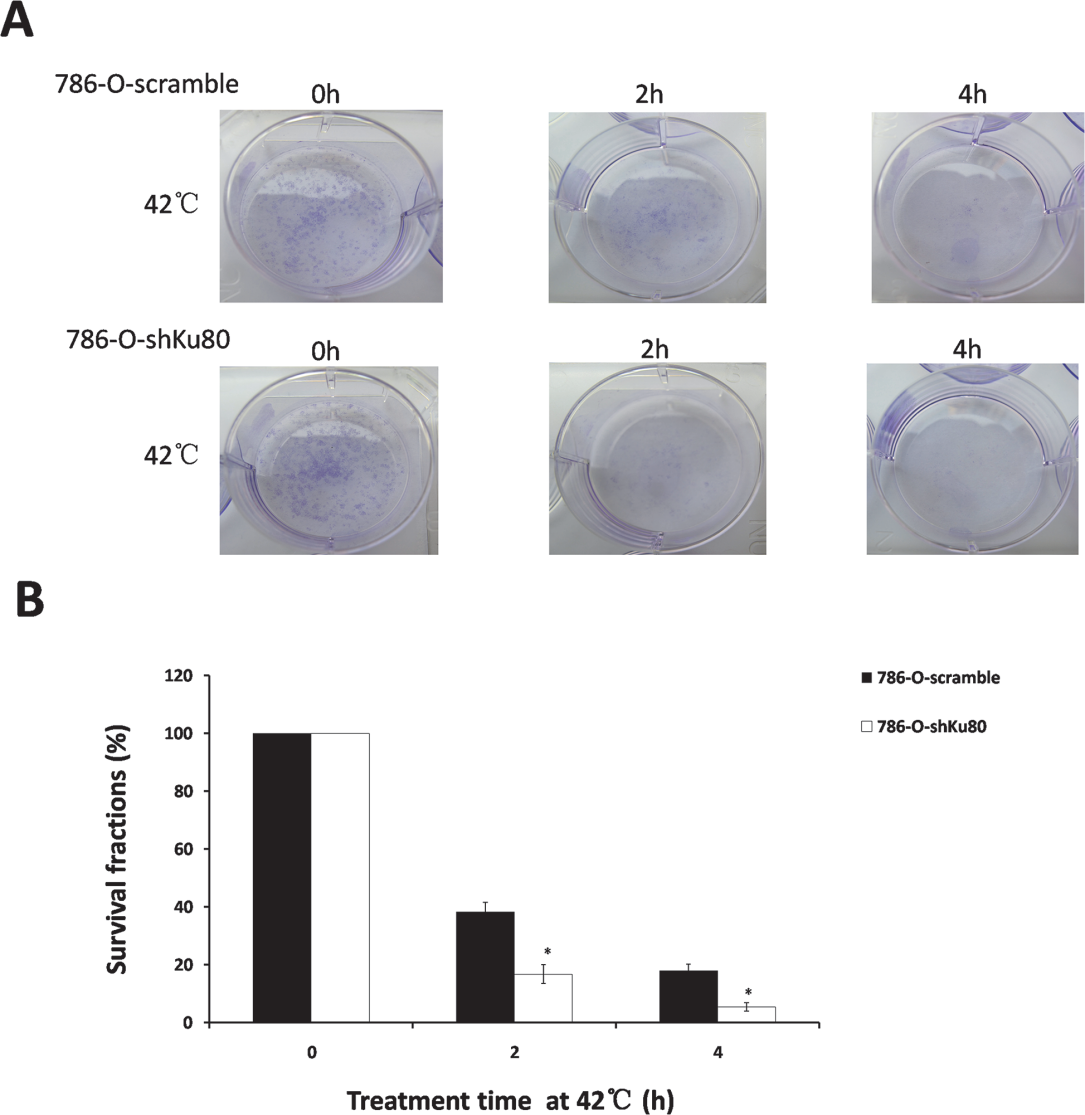
Effect of Ku80 expression on heat-sensitivity under hyperthermia. 786-O-shKu80 and 786-O-scramble cells were subjected to 42°C for the indicated amount of time. Survival fractions were measured by colony formation assay after hyperthermia. **A:** Images showing colony formation assay. **B:** The histogram shows the result from A (%). *P<0.05 compared to control. Each date point is the mean of three independent experiments.

To confirm the heat sensitivity of 786-O-shKu80 cells and 786-O-scramble cells, we performed colony formation assay after hyperthermia for various times and found that the surviving fractions were reduced in 786-O-shKu80 cells compared with 786-O-scramble cells at each time point, and at 2h and 4h, the surviving fraction of 786-O-shKu80 cells was decreased by 21.7% and 12.7%, respectively (Fig 5). It can thus be seen that 786-O-shKu80 cells are more heat sensitive than 786-O-scramble cell; in other words, reduction of Ku80 expression makes the 786-O cells more sensitive to heat.

### Ku80 mediates hyperthermia-induced apoptosis through influencing the cell cycle distribution

**Fig 6:**
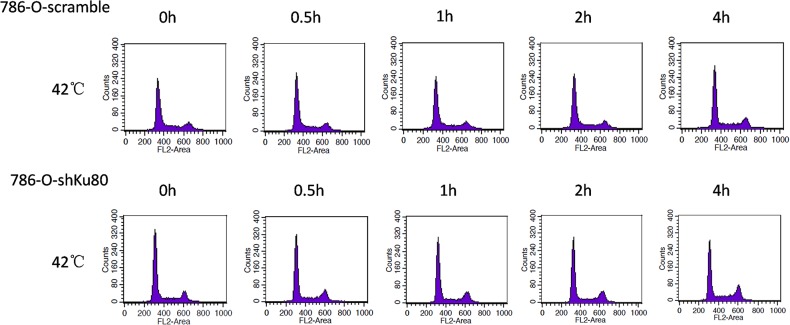
Effect of Ku80 expression on cell cycle distribution under hyperthermia. 786-O-shKu80 and 786-O-scramble cells were subjected to 42°C for the indicated amount of time. Then the cell cycle distribution was measured immediately after hyperthermia. Images showing flow cytometric analysis of cell cycle distribution.

The above findings suggest Ku80 plays an important role in hyperthermia-induced apoptosis. In order to detect the possible apoptotic pathways mediated by Ku80, 786-O-shKu80 and 786-O-scramble cells were also exposed to 42°C for various times and then the percentage of cells in different cell cycle phases was determined by the flow cytometry method (Fig 6). The results indicated that compared to 786-O-scramble cells, a significant G2/M phase cell cycle checkpoint accumulation was observed in 786-O-shKu80 at each time point, and at 1h, 2h and 4h, the percentage of G2/M phase cells was increased by 5.38%, 6.08% and 8.30%, respectively (Tables 3 and 4). These results show that low-expression of Ku80 can result in G2/M phase arrest under the hyperthermia in 786-O cells, demonstrating that Ku80 mediates hyperthermia-induced apoptosis through influencing cell cycle in G2/M phase.

**Table 3:** Cell cycle detection in 786-O-scramble at 42°C for the indicated amount of time.

Treatment time (h)	0	0.5	1	2	4
**G0/G1**	68.17±1.35	67.61±1.57	66.95±2.01	67.14±2.20	62.17±2.38
**S**	18.01±1.26	18.61±1.51	20.34±1.79	20.56±2.13	22.44±2.35
**G2/M**	13.82±1.09	13.78±1.83	12.71±2.12	12.30±2.05	15.39±2.17

**Table 4:** Cell cycle detection in 786-O-shKu80 at 42°C for the indicated amount of time.

Treatment time (h)	0	0.5	1	2	4
**G0/G1**	69.40±1.45	66.53±1.32	66.93±1.50	63.52±2.23	55.37±2.44
**S**	13.67±1.38	14.44±1.59	14.98±1.62	18.10±2.01	20.94±2.07
**G2/M**	16.93±1.23*	19.03±1.36*	18.09±1.74*	18.38±2.18*	23.69±2.38*

*P<0.05 compared to control

## Discussion

Hyperthermia has become the fifth method of therapy after surgery, chemotherapy, radiation and biological therapy, and plays an important role in multidiscipline therapy for cancer. Hyperthermia like radiofrequency ablation (RFA) is an accepted alternative to surgery in selected patient populations with RCC; specifically, in patients with tumors smaller than 4 cm who are not surgical candidates [31]. It has shown promising results due to its principal effect to induce necrosis and apoptosis of cancer cells. Therefore, understanding the mechanisms of hyperthermia-induced apoptosis of cancer cells will contribute to treat RCC (especially advanced RCC) and other cancers.

Apoptosis is a programmed cell death, which occurs in both physiological and pathological conditions and plays an important role in the regulation of tissue development. Tumor cell apoptosis can be induced by DNA-damaging treatments like chemotherapy and radiotherapy, and physiological stress conditions such as hyperthermia [32, 33]. There have been extensive studies on the signaling pathways that mediate the apoptotic effects of hyperthermia. For example, hyperthermia induces apoptosis mainly through reactive oxygen species (ROS) generation, and a likely source of elevated ROS production is the mitochondria [34]. In addition, hyperthermia can also alter the expression of the Bax and Bcl-2 genes, where such changes are dependent on the sensitivity of cell lines to hyperthermia [35]. Kajihara et al. also proposed a possible heat-induced p53-dependent signal transduction pathway for apoptosis, they found that p53 gene status is closely associated with heat-induced apoptosis, and with the expression of apoptosis-inhibitory proteins, such as Bcl-2, Bcl-xL and IL-6 [36]. Furthermore, hyperthermia-induced increase in intracellular Ca^2+^ ion concentration is also thought to be involved in cell death [37]. In present study we found that hyperthermia inhibited the release of Ku80 in renal carcinoma 786-O cells followed by the activated spontaneous apoptosis, and low-expression of Ku80 could result in apoptosis and G2/M phase arrest directly under the hyperthermia. Our findings imply that the molecular mechanisms of hyperthermia-induced apoptosis may include: hyperthermia attenuates the expression of Ku80, and then reduction of Ku80 expression induces apoptosis through arresting the cell cycle in G2/M phase.

In this study, we found hyperthermia inhibited the expression of Ku80, but no Ku70 expression in 786-O cells. Ihara et al. found hyperthermia can suppress both Ku70 and Ku80 expression in hybrid cells containing a fragment of human chromosome 8 in scid mouse cells [30]. The different results may be due to the cell lines derived from different species and tissues in each experiment.

It is well known that Ku80 is an important DNA repair protein in the NHEJ pathway, and it also involved in other cellular processes, such as telomere maintenance, regulation of apoptosis, tumor suppression and gene regulation. Previous studies have shown that the inhibition of Ku80 expression resulted in the induction of apoptosis in embryonic stem cells, pre-B cells and human HCT116 colon cancer cells [38, 39]. These results indicate that suppressing apoptosis is another function of Ku80, which can help us explain our research results—the apoptotic rates of 786-O-shKu80 cells were significantly higher than 786-O-scramble cells under the same condition of hyperthermia. We also demonstrate that suppression of Ku80 can result in G2/M phase arrest under the hyperthermia in 786-O cells. Consistent with our results, Nussenzweig et al. reported that a prolonged G2/M phase was observed in fibroblasts from Ku80-deficient mouse embryos [40]. Munoz et al. also showed a novel role for Ku antigen in the G2 and M phases of the cell cycle, one that appears independent of its participation in DSB repair by DNA-PK [41].

Our results demonstrated that hyperthermia suppressed the Ku80 expression, which made the 786-O cells more susceptible to hyperthermia. G2/M phase arrest caused by reducing of Ku80 expression may contribute to a new perspective to explain this phenomenon. Meanwhile, some studies have shown that heat, like X-rays, may lead to the induction of DSBs [42–45], of which Takahashi et al. proposed that heat-induced cell killing may be dependent or associated with DSB formation in mammalian cells, suggesting that heat induces DSB formation through the denaturation and dysfunction of heat-labile repair proteins such as DNA polymerases; Okamoto et al. reported that hyperthermia can induce DSBs, and transfection of NBS1-siRNA into 8305c cells enhances heat sensitivity and the frequency of apoptosis through caspase pathway, which suggests that heat sensitisation may result from the NBS1-siRNA mediated suppression of DNA repair. It is generally known that the Ku heterodimer (Ku70/Ku80) plays a central role in DSBs repair. Therefore, the decline of Ku80 expression may also increase heat-sensitivity in 786-O cells through the suppression of heat-induced DSB repair.

Hyperthermia is regarded as a potent radiosensitizer and chemosensitizer as its important role in inhibiting the repair of DSBs in numerous studies. Our findings that hyperthermia inhibits Ku80 expression might help understand the process of DSBs repair induced by hyperthermia. It is the reduction of Ku80 expression induced by hyperthermia that inhibits the repair of DSBs and results in radio- and chemosensitization ultimately.

In conclusion, our data in the present study demonstrate for the first time that hyperthermia can inhibit the expression of Ku80, and reduction of Ku80 expression induces apoptosis directly through arresting the cell cycle in G2/M phase in 786-O cells. These results point to a new perspective of hyperthermia cytotoxicity, and might provide the scientific basis for developing cancer therapy with hyperthermia treatment as a single agent or as a part of rational combinations in RCC.
